# Ensuring a culturally sensitive, patient centred approach to podiatric management of high risk clients from a refugee background

**DOI:** 10.1186/1757-1146-8-S2-P11

**Published:** 2015-09-22

**Authors:** Lauren Farnsworth, Kathleen O'Brien

**Affiliations:** 1 Barwon Health, Corio Community Health Centre, Corio, Victoria 3214, Australia

**Keywords:** Refugee, cultural sensitivity, Podiatry

## Background

Over recent years an increasing number of complex, high risk clients from a newly arrived refugee background have presented for podiatric intervention within Barwon Health. Whilst the clinical presentations alone have proved unusual and challenging, a range of cultural and psycho-social factors have required negotiation in order to obtain optimal patient outcomes.

## Process

This presentation aims to share details relating to the experience of managing 3 particularly challenging refugee cases and the successful strategies that were employed to maximise client outcomes and demonstrates the expertise which has been developed in this niche area of podiatry service delivery.

## Findings

Podiatrists employed a range of culturally sensitive strategies to effectively manage clients presenting with Hansen's disease, Rickets and a Diabetes foot wound/ calcaneal fracture. These included the delivery of group education programs in languages other than english, client advocacy, working closely with family members and interpreters, negotiating health beliefs and customs, obtaining funding and problem solving around social factors that were impacting on foot health.

## Conclusions

Employing a patient centred, culturally sensitive approach was essential in obtaining the trust and engagement of clients from a refugee background. To maximise client outcomes, these strategies should become an integral component of the approach of all podiatrists who work with culturally and linguistically diverse populations.

